# Gut microbiome restructuring in laryngeal squamous cell carcinoma identifies stable microbial biomarkers with diagnostic potential

**DOI:** 10.3389/fonc.2026.1788705

**Published:** 2026-04-22

**Authors:** Jiaming Zhang, Jingtai Zhi, Jianxin Li, Li Li, Shengchi Zhang, Juntao Niu, Wei Wang

**Affiliations:** 1 Department of Otorhinolaryngology Head and Neck Surgery, Tianjin First Central Hospital, Tianjin, China; 2 Institute of Otolaryngology of Tianjin, Key Laboratory of Auditory Speech and Balance Medicine, Tianjin, China; 3 Key Medical Discipline of Tianjin (Otolaryngology), Quality Control Centre of Otolaryngology, Tianjin, China; 4 Graduate School, Tianjin Medical University, Tianjin, China; 5 LC-Bio Technology Co., Ltd., Hangzhou, China; 6 Department of Otorhinolaryngology, Head and Neck Surgery, The Second Hospital of Tianjin Medical University, Tianjin, China

**Keywords:** 16S rRNA sequencing, biomarker, functional pathways, gut microbiota, laryngeal squamous cell carcinoma, microbial dysbiosis

## Abstract

**Background:**

Alterations in the gut microbiota have been reported in various malignancies, but its role in laryngeal squamous cell carcinoma (LSCC) remains unclear.

**Methods:**

This retrospective study included 101 patients undergoing laryngeal surgery (46 benign, 55 malignant). Preoperative fecal samples were collected and subjected to 5R 16S rRNA sequencing. Sequencing data were processed using DADA2 and QIIME2, followed by α/β diversity analysis, differential abundance detection (Wilcoxon test, LEfSe, random forest), and LASSO regression. Functional pathway differences were inferred using PICRUSt2.

**Results:**

There were no significant differences in α diversity metrics between groups, whereas β diversity analysis revealed significant separation between Benign and LSCC (PERMANOVA, P<0.01). Distinct community composition differences were observed: Malignant cases showed enrichment of genera such as *Streptococcus* and *Lactobacillus*, while Benign cases exhibited enrichment of genera including *Bacteroides* and *Lachnospira*. Multimethod integration identified 17 core bacterial genera, which were further refined via LASSO regression to select a stable set of genera (e.g., *Streptococcus*, *Eubacterium*, *Lachnospira*) capable of reliably distinguishing benign from malignant cases. The logistic regression model based on stable genera demonstrated excellent diagnostic performance (AUC > 0.8), particularly in distinguishing benign from LateLSCC. Functional prediction revealed pathway imbalances: malignant cases showed enrichment in cell wall and amino acid synthesis pathways, while benign cases favored vitamin and steroid metabolism pathways.

**Conclusion:**

LSCC patients exhibit structural remodeling of their gut microbiota, characterized by distinct taxonomic and functional alterations. Stable microbial signatures holding potential as a foundation for the future development of non-invasive diagnostic and staging biomarkers, though their clinical translation necessitates further large-scale validation.

## Introduction

Head and neck squamous cell carcinoma (HNSCC) ranks as the sixth most common malignant tumor globally, with a five-year survival rate below 60%, posing a severe threat to human health (1–3).Smoking, alcohol consumption, and human papillomavirus (HPV) infection are recognized primary risk factors; however, these traditional factors fail to fully explain its complex pathogenesis and significant clinical heterogeneity. Laryngeal squamous cell carcinoma (LSCC), a significant subtype of HNSCC, exhibits high recurrence and metastasis rates with poor clinical prognosis, necessitating exploration of novel pathogenic mechanisms and non-invasive diagnostic approaches (4, 5).

Recent microbiome studies have progressively revealed the critical role of gut microbiota in tumorigenesis and progression (6, 7). Previous studies have primarily focused on gastrointestinal tumors, such as esophageal squamous cell carcinoma and colorectal cancer, revealing significant alterations in gut microbiota composition: a marked decrease in probiotic genera like *Faecalibacterium* and *Roseburia*, alongside an increase in potentially pathogenic bacteria such as *Lactobacillus* and *Romboutsia*. These microbial dysbiosis not only correlates with local metabolic imbalance and chronic inflammation but may also promote tumorigenesis and progression through immune dysregulation (7–9). Notably, recent evidence further indicates that gut microbiota dysregulation extends beyond gastrointestinal cancers, influencing distant organs via the “gut-organ axis” (10): gut microbiota can affect brain tumor development through immune and inflammatory pathways, regulate liver cancer progression via the enterohepatic circulation, increase breast cancer risk through estrogen metabolism imbalance, and modulate the lung cancer immune microenvironment via the “gut-lung axis” (8) (11–13).These findings suggest that gut microbiota may serve as a systemic regulatory factor for multiple malignancies, including laryngeal cancer.

At the mechanistic level, gut microbiota can remodel the tumor microenvironment through multiple pathways, including short-chain fatty acid (SCFA) metabolism, lipopolysaccharide (LPS)-mediated chronic inflammation, immune checkpoint regulation, and metabolic signaling pathways (14, 15). For instance, butyrate-producing bacteria enhance antitumor immunity, whereas LPS from Gram-negative bacteria may promote immune evasion. Functional prediction studies also reveal that tumor-associated microbiota frequently enriches amino acid metabolism and cell wall synthesis pathways, whereas healthy individuals favor vitamin and energy metabolism. This metabolic functional reshaping may manifest early in carcinogenesis.

However, to date, studies on the gut microbiota characteristics of laryngeal squamous cell carcinoma (LSCC) patients remain scarce. Existing literature predominantly focuses on oral microbiota or gastrointestinal tumors, with virtually no systematic investigations into LSCC-associated gut microbiota composition, functional pathways, and their clinical significance (6, 16, 17). This knowledge gap limits our understanding of the mechanisms underlying laryngeal cancer development and hinders the translation of microbiological discoveries into clinical diagnostic and staging tools.

## Method

### Study population

Pathological findings were categorized into the Benign group (46 cases) and the malignant (CA) group (55 cases), comprising 34 cases of early LSCC and 21 cases of LateLSCC. Benign lesions were specifically chosen as the control group to simulate the real-world clinical dilemma of differentiating between precancerous/benign growth and malignant transformations in the larynx. To minimize dietary confounding factors, all fecal samples were collected during the preoperative hospitalization period, during which patients were provided with relatively standardized hospital meals. Clinical and pathological data were collected from medical records, with diagnoses confirmed by histopathological examination. Specifically, p16 expression was evaluated using immunohistochemistry (IHC) with a monoclonal antibody (e.g., clone E6H4). According to standardized scoring criteria for head and neck cancers, p16 positivity was defined as strong and diffuse nuclear and cytoplasmic staining in 70% of tumor cells. This variable was included to explore whether the gut microbiome profiles differ between p16-positive and p16-negative LSCC patients, given the profound biological differences often associated with p16/HPV status in upper respiratory tract malignancies (18). Exclusion criteria included: (1) patients with immune/genetic disorders, pregnancy, or coagulation disorders; (2) patients who had used systemic antibiotics, immunosuppressants, or steroids within 12 weeks prior to screening ; (3) patients with malignancies other than HNSCC (e.g., lymphoma or metastatic cancer). A total of 12 patients were excluded, leaving 101 patients whose samples were used for subsequent analysis. Each patient underwent physical examination by two physicians, with age, sex, smoking history, alcohol consumption, and betel nut chewing history recorded. Alcohol consumption was defined as the intake of at least one standard alcoholic beverage per week for at least six months. Notably, as this study was conducted in Northern China (Tianjin), where betel nut chewing is not a prevalent habit, none of the participants in this cohort reported a history of betel nut use. LSCC patients received neck and chest imaging, and TNM staging was determined based on imaging, pathology, and biopsy results according to the American Joint Committee on Cancer (AJCC) Cancer Staging Manual, 8th Edition. All participants provided informed consent, and this study was approved by the local ethics committee (ID: 2023DZX46).

### Sample processing

To evaluate changes in the fecal microbiome during LSCC tumorigenesis, we collected fecal samples for 5R 16S rRNA sequencing. Compared to conventional 16S rRNA gene sequencing, this technology enhances the sensitivity and specificity of 16S sequencing for microbial identification. It also enables detection of trace microbial populations in samples with low microbial content, such as tumor tissues and bodily fluid specimens. To ensure hygiene, sterile gloves must be worn during collection. Use a sterile toothpick or fecal sampler to obtain mid-layer samples from the inner portion of the stool, avoiding contamination from surface intestinal mucosal cells exposed to air and potential degradation of bacterial DNA. Collected samples are then aliquoted into 2 mL sterile Eppendorf tubes, each containing 0.5–2 g of stool. Each sample must be aliquoted or duplicated into 2–3 tubes for backup. After proper packaging, samples are rapidly frozen in liquid nitrogen and stored long-term at -80 °C or in liquid nitrogen. Blank collection tubes exposed for 30 seconds in the sampling area serve as negative controls. Preliminary testing confirmed sample quality and method feasibility. All procedures were conducted in a sterile operating room.

### 5R 16S rRNA amplicon sequencing

Preoperative fecal samples were processed to extract microbial genomic DNA. This study utilized a 5R 16S sequencing strategy as previously described by Nejman et al. (Science, 2020). Instead of conventional single-region amplification (such as V3–V4), this method involves multiplex PCR amplification of five non-contiguous variable regions (V2, V3, V5, V6, and V8) of the 16S rRNA gene. By capturing approximately 68% of the full-length 16S sequence, this approach significantly enhances taxonomic resolution and species-level identification compared to standard V3-V4 protocols. The specific primer pairs used for multiplexing were: V2 (27F/338R), V3 (341F/518R), V5 (784F/939R), V6 (967F/1046R), and V8 (1249F/1407R). PCR products were purified and sequenced on the Illumina NovaSeq 6000 platform (PE250). To reconstruct the multi-region data, raw sequences were processed using the Short MUltiple Regions Framework (SMURF) to ensure high-accuracy taxonomic assignment against the SILVA (v138.1) and NT-16S (v2022.10) databases (19, 20).

### Quality control and decontamination

Exclude samples with insufficient effective read counts after pooling (≥20,000 reads or using the Shannon dilution curve platform as threshold). Remove mitochondrial, chloroplast, and unannotated ASVs; filter ASVs with global abundance <0.01% or sample prevalence <5%. Identify and remove contamination using decontam (prevalence+frequency, threshold 0.1–0.5) in conjunction with negative controls.

### Normalization and transformation

α-diversity (Shannon, Simpson, Chao1, ACE) was calculated on ASV tables rarefied to a uniform depth. Taxonomic assignment was performed at the ASV level. For all downstream comparative analyses—including differential abundance testing and diagnostic modeling—individual ASV counts were aggregated to the genus level by summing the abundances of all ASVs assigned to the same genus. This aggregation level was chosen to provide a balance between high taxonomic resolution and statistical robustness for identifying clinical biomarkers. β-diversity was determined using Bray–Curtis distance based on the relative abundance matrix; for Aitchison distance, zeros were first replaced with zCompositions::cmultRepl, followed by Centered Log-Ratio (CLR) transformation using compositions::clr. Visualization was performed via PCoA (Bray–Curtis) and PCA (CLR matrix), plotting 95% confidence ellipses.

### Community structure differences

PERMANOVA (vegan:adonis2, 9,999 permutations), model: Distance ~ Group + Age + Gender + Smoking +Alcohol+ Batch; Test homogeneity of dispersion using betadisper/permutest.

### Community composition visualization

Phylum- and genus-level stacked bar charts and Top 30 genus bubble plots (low abundance <0.1% merged as “Others”), based on phyloseq/ggplot2.

### Differentially abundant genera screening (multiple methods consensus)

**Figure 1:**
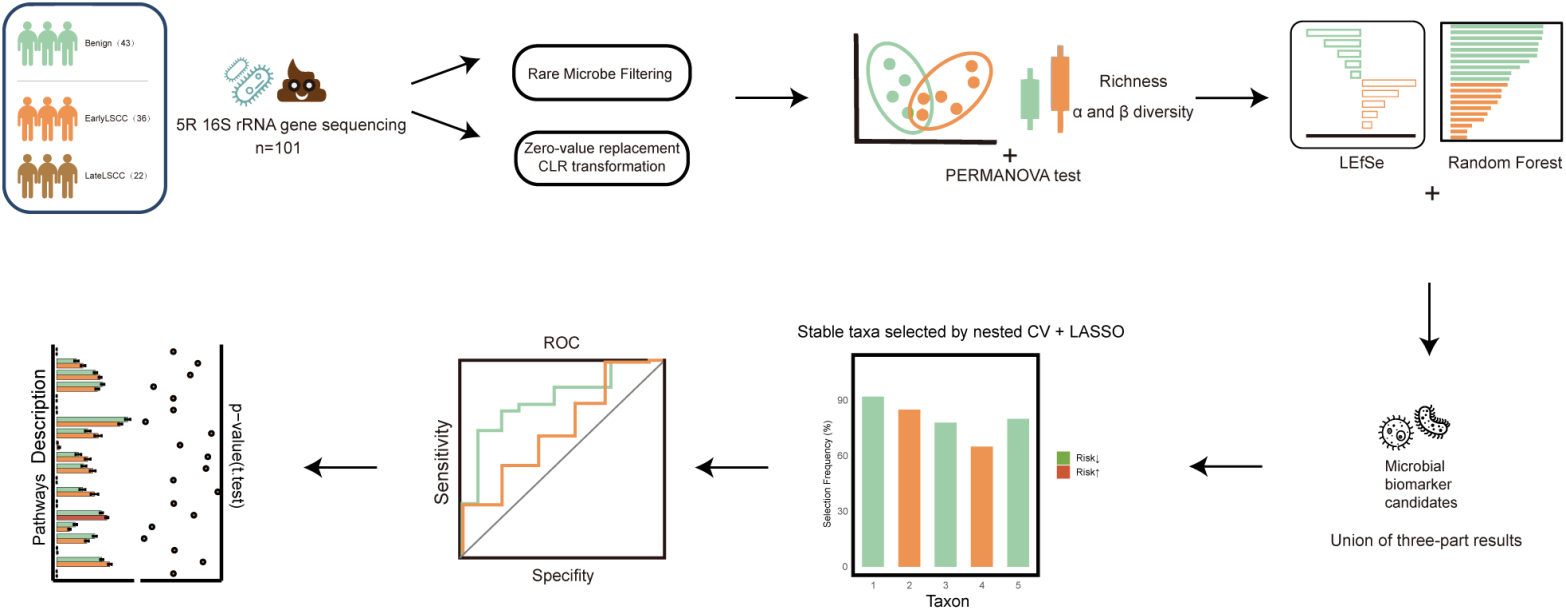
Study workflow for the identification and validation of gut microbial biomarkers in laryngeal squamous cell carcinoma (LSCC). The analytical pipeline began with the collection of preoperative fecal samples from 101 participants, categorized into Benign (n = 43), EarlyLSCC (n = 36), and LateLSCC (n = 22) cohorts. Following 5R 16S rRNA gene sequencing, data underwent rare microbe filtering, zero-value replacement, and Centered Log-Ratio (CLR) transformation. Alpha and beta diversity analyses (PERMANOVA) and multi-method taxonomic screening (LEfSe and Random Forest) were performed to identify microbial biomarker candidates. A stable set of genera was then selected through 100-repeated nested cross-validation (Nested CV) and LASSO regression. The final outputs included the construction of a diagnostic model (ROC analysis) and the characterization of functional pathway alterations.

(1) Wilcoxon signed-rank test (two-tailed), reporting Cliff’s delta; BH-corrected, threshold *q* < 0.1 and |log_2_ difference|>0.5. (2) LEFSe (LDA≥2.5, P<0.05), with staging as subclasses when necessary. (3) Random Forest (ranger): Features: genus-level CLR or relative abundance. 1,000 trees. Additionally, to ensure the robustness of our taxonomic findings as shown in the workflow (**Figure 1**), we validated these results using ANCOM-BC and ALDEx2 algorithms, which account for the compositional nature of microbiome data, with significance defined at q < 0.05. Output: Permutation importance and Gini decay. Extract Top 20. (4) Identification of Core Candidate Genera: A consensus-based strategy was implemented to identify robust microbial biomarkers. A genus was defined as a “core candidate” if it met either of the following rigorous criteria: (i) Strict Intersection: it was simultaneously identified as significant by all three methods (Wilcoxon rank-sum test *q* < 0.1, LEfSe LDA score ≥ 2.5, and ranked within the Top 20 of Random Forest importance); or (ii) High-Confidence Overlap: it showed consistent significant enrichment in the same group across both Wilcoxon and LEfSe tests while maintaining consistent directionality in the Random Forest model. This multi-method integration ensures that the selected genera are not artifacts of a single statistical approach but represent biologically stable features for downstream diagnostic modeling.

### LASSO screening

Use core candidate genus CLR abundance as independent variable, glmnet logistic regression (α=1). Nested cross-validation: Inner 5-fold parameter tuning, outer 5-fold evaluation, repeated 100 times. To address the inherent challenges of collecting rare LSCC clinical samples and to compensate for the current lack of an independent multi-center validation cohort, we implemented a rigorous 100-repeated nested cross-validation (Nested CV) scheme. This framework—comprising an inner loop for parameter tuning and an outer loop for unbiased performance evaluation—ensures that the diagnostic model is tested on data completely independent of the feature selection process, thereby providing a highly conservative and robust estimate of its generalizability. Record feature selection frequency; define stable genera as those with selection frequency >50% and consistent coefficient direction.

### Diagnostic model

Construct a core logistic regression model using stable genera; add age, sex, smoking status, and stage to the multivariate model. Evaluate performance via stratified 5-fold CV × 20 replicates: primary metric ROC-AUC, supplemented by PR-AUC, sensitivity, specificity, and Youden’s index. AUC 95% CI calculated using the DeLong method (pROC), with 1,000 bootstrap samples for optimistic calibration and reporting of calibrated AUC. Calibration curves and Hosmer–Lemeshow tests performed; Platt scaling or rms:calibrate recalibration applied when necessary. Submodels for “Benign vs. LateLSCC” validated using identical methods.

### Function prediction

Perform SEPP placement and HSP for ASV sequences using PICRUSt2 (≥v2.5) to obtain EC/KO and MetaCyc pathway abundance; exclude subjects with NSTI > 2.0; standardize by total sample size or correct using pathway copy number. Intergroup differences analyzed using Wilcoxon test with BH correction (*q* < 0.1); multivariate validation via MaAsLin2 (fixed effect: group; covariates: age, sex, smoking status, alcohol consumption, batch). Genus–pathway Spearman correlations calculated using CLR relative abundances of genera and pathways, reporting *ρ* and *q* values; the strength of Spearman’s correlation rho was interpreted as follows: |*ρ*|≥0.6 was defined as a strong correlation, 0.3≤|*ρ*|< 0.6 as a moderate correlation, and *ρ* < 0.3 as a weak correlation. Associations reaching both statistical significance (*q* < 0.05) and at least moderate correlation strength (|*ρ*|≥0.3) were prioritized for biological interpretation.” Displayed “Top 30 Genera × Significant Pathways” heatmaps, grouped by functional categories (cell wall/amino acids/vitamins-coenzymes/nucleotides, etc.), comparing trajectories across different stages.

### Statistical analysis

All data analyses were performed using R version 4.2.2 (2022–10–31). To address potential confounding effects of baseline clinical characteristics—particularly the significant age difference observed between groups—age, gender, smoking status, and alcohol consumption were included as covariates in all major downstream analyses. This includes PERMANOVA for community structure, MaAsLin2 for multivariate functional validation, and the construction of the final diagnostic model, ensuring that the identified microbial signatures are independently associated with LSCC. All statistical tests employed two-tailed significance testing, with P < 0.05 indicating statistically significant differences. Normally distributed quantitative data are presented as mean ± standard deviation (Mean ± SD). Intergroup comparisons were performed using one-way analysis of variance (ANOVA). Non-normally distributed quantitative data are presented as median and interquartile range [M (Q1, Q3)]. and compared between groups using the Kruskal-Wallis H test. Count data were expressed as number and proportion [n (%)]. For unordered categorical data, intergroup comparisons were performed using Pearson’s χ² test or Fisher’s exact test; for ordered categorical data, the Kruskal-Wallis H test was used. For multiple comparisons, Scheffe’s method was applied to normally distributed quantitative data, Bonferroni’s method to non-ordered categorical data, and Nemenyi’s method to ordered categorical data. Simultaneously, standardized mean differences (SMD) were used to compare intergroup variations. Generally, SMD < 0.10 indicates acceptable equivalence between groups, 0.10–0.34 denotes minor differences, 0.35–0.64 indicates moderate differences, 0.65–1.19 signifies substantial differences, and SMD ≥ 1.20 reflects very large differences between groups (21).

### Data availability

The raw sequencing data generated in this study have been deposited in the Genome Sequence Archive (GSA-Human: HRA013872) at the National Genomics Data Center, Beijing Institute of Genomics, Chinese Academy of Sciences, under BioProject accession number PRJCA048473.These data are publicly available at https://ngdc.cncb.ac.cn/gsa-human (22, 23).

### Software and environment (detailed R and Python package versions for reproducibility)

QIIME2: 2023.5, PICRUSt2: v2.5.0, R: v4.2.2 (Platform: Ubuntu 20.04 LTS, 64-bit), Python: v3.9.16; Primary R packages: phyloseq v1.42.0, vegan v2.6-4, ggplot2 v3.4.4, ranger v0.15.1, caret v6.0-94, glmnet v4.1-8, pROC v1.18.0, rms v6.7-0, MaAsLin2 v1.12.0, zCompositions v1.5.0-2, compositions v2.0-6, decontam v1.20.0; Python libraries: numpy v1.23.5, pandas v1.5.3, scikit-learn v1.2.2, matplotlib v3.7.2, seaborn v0.12.2.

## Result

### Baseline demographic and clinical characteristics

This study included 101 patients, comprising 46 cases of benign lesions, 34 cases of EarlyLSCC, and 21 cases of LateLSCC. The baseline clinical and pathological characteristics of the three patient groups are presented in **Supplementary Table 1**. Significant differences in age distribution were observed among the groups (*F* = 4.147, *P* = 0.019). Further multiple comparisons revealed a significant difference between the Benign group and the LateLSCC group (*P* = 0.028), while no statistically significant differences were found between the Benign group and the EarlyLSCC group (*P* = 0.181) or between the EarlyLSCC and LateLSCC groups (*P* = 0.570). Gender distribution showed no significant differences among the three groups (*P* = 0.542). Smoking status (*P* = 0.435) and alcohol consumption (*P* = 0.832) were evenly distributed across the groups, with no statistically significant differences. Pack-years of smoking showed an increasing trend from the Benign to the LSCC groups, but the difference was only marginally significant (*P* = 0.060). Significant differences were observed in tumor staging-related indicators. T staging showed significant differences among the three groups (χ²=86.582, *P* < 0.001), and N staging also exhibited significant differences (χ²=92.904, *P* < 0.001). Multiple comparisons further indicated a statistically significant difference in N staging between EarlyLSCC and LateLSCC groups (*P* = 0.016). M staging also showed significant differences between the Benign group and Early/LateLSCC groups (χ²=101.000, *P* < 0.001). Overall TNM staging differences were most pronounced (χ²=202.000, *P* < 0.001), with early-stage cases predominantly in stages I–II and LateLSCC cases primarily in stages III–IV. Immunohistochemical results showed that the p16 positivity rate was slightly higher in the Benign group (21.7%) than in EarlyLSCC (11.8%) and LateLSCC (4.8%), but the difference was not statistically significant (*P* = 0.204). In summary, significant differences existed among the three groups in age and tumor stage, while factors such as gender, smoking, and alcohol consumption were evenly distributed across groups. This provides a comparable foundation for subsequent gut microbiota differential analysis.

### Microbial diversity analysis

**Figure 2:**
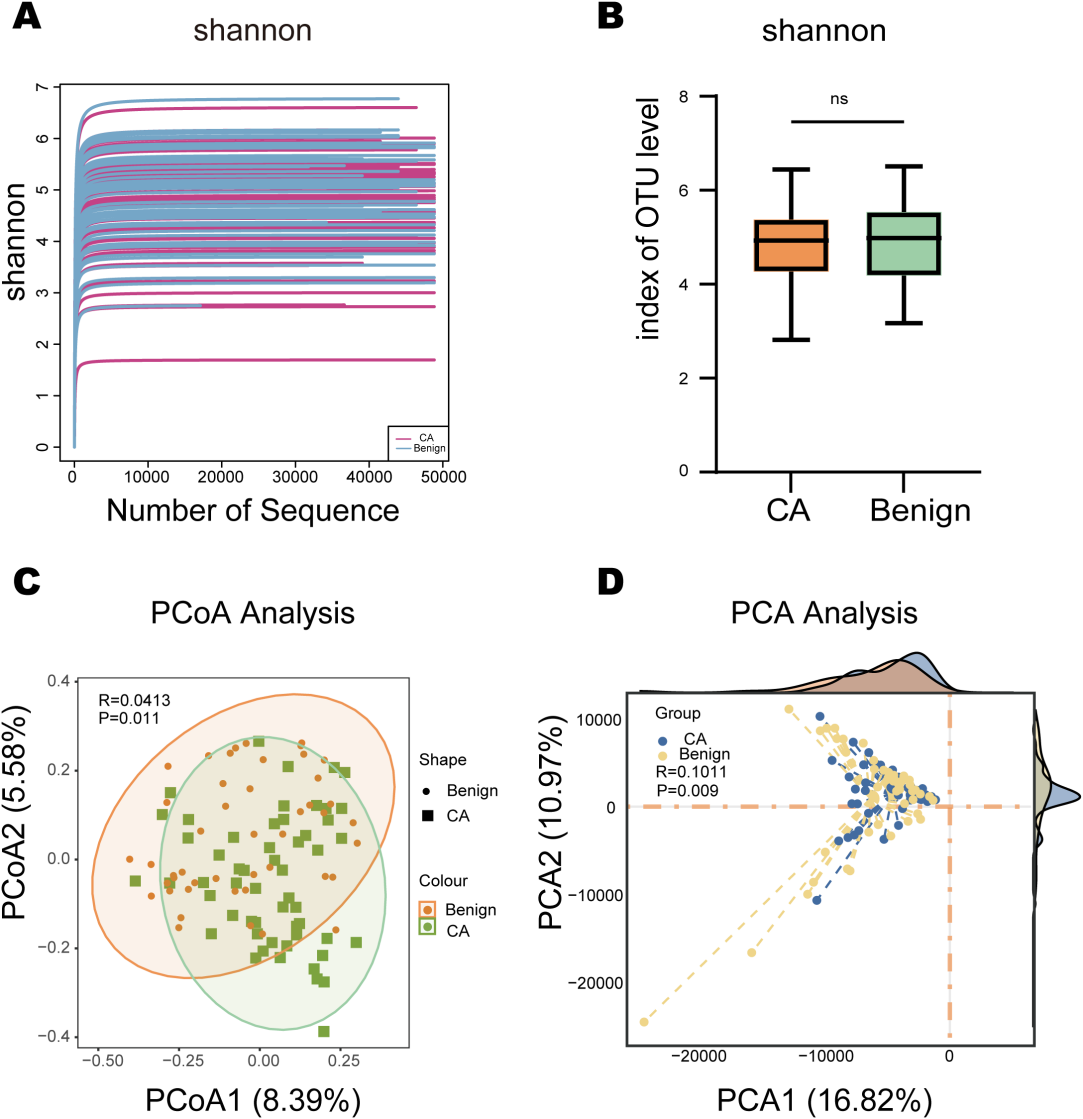
Gut microbial diversity analysis in LSCC and Benign groups. **(A)** Shannon dilution curves for all samples illustrating sufficient sequencing depth. **(B)** Boxplot of Shannon index comparing alpha diversity between CA and Benign groups (ns, not significant). **(C)** Principal Coordinate Analysis (PCoA) based on Bray-Curtis distance revealing significant community separation between groups (PERMANOVA, P = 0.011). **(D)** Principal Component Analysis (PCA) based on Aitchison distance (CLR-transformed) further validating the divergence in community structure (P = 0.009).

To validate sequencing depth, Shannon dilution curves for all samples reached a distinct plateau phase (**Figure 2A**). Other α-diversity metrics—including species coverage, Simpson’s index, Chao 1 index, and observed species richness—exhibited similar saturation trends with no significant differences between CA and Benign groups (**Supplementary Figures 1A, B**). Boxplots of Shannon indices similarly indicated no significant intergroup differences (**Figure 2B**, *P*>0.05). After stratification by Benign, EarlyLSCC, and LateLSCC, α-diversity boxplots revealed selective pairwise differences between stages (**Supplementary Figure 1C**).Regarding β-diversity, Bray-Curtis principal coordinate analysis revealed separation between the CA and Benign groups, with PERMANOVA indicating significant differences (R = 0.0413, *P* = 0.011; **Figure 2C**). Principal component analysis further validated this pattern (R = 0.1011, *P* = 0.009; **Figure 2D**). β-diversity analysis based on disease stage further revealed distinct clustering among the three groups. PCoA analysis showed significant differences among the three groups (overall PERMANOVA R = 0.1027, *P* = 0.001), consistent with PCA results (R = 0.10257, *P* = 0.001; **Supplementary Figure 1D**). *Post-hoc* pairwise PERMANOVA comparisons further revealed significant community divergence between the Benign and EarlyLSCC groups (R = 0.065, *P* = 0.012), between the Benign and LateLSCC groups (R = 0.118, *P* = 0.002), and between the Early and LateLSCC stages (R = 0.052, *P* = 0.038). In summary, while α-diversity indices were comparable between groups, β-diversity analysis demonstrated significant clustering separation between the Benign and LSCC, as well as distinct community structures across the various clinical stages of LSCC.

### Microbial community composition

**Figure 3:**
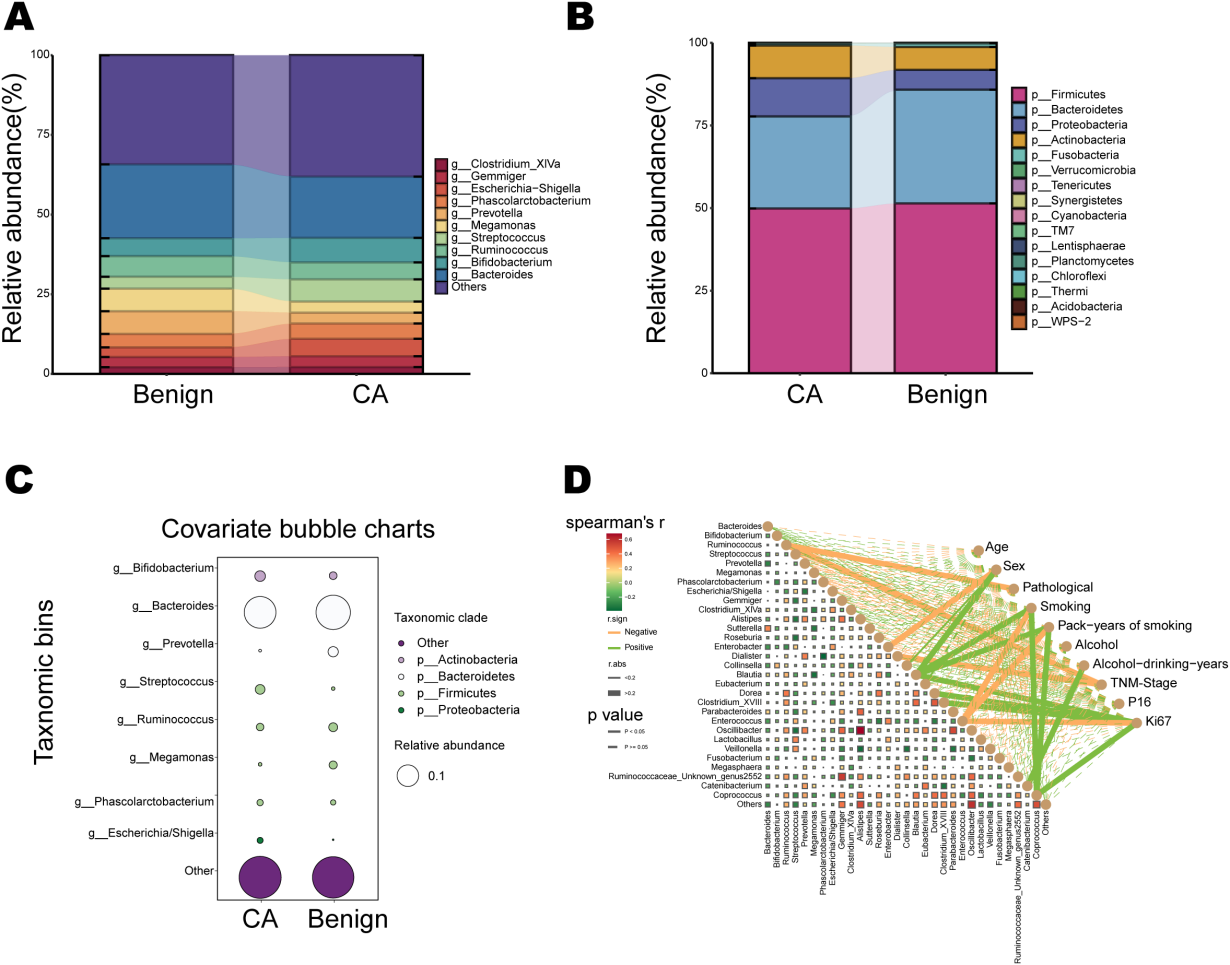
Gut microbiota composition and its correlation with clinical characteristics. **(A, B)** Stacked bar charts showing the relative abundance of dominant bacterial genera **(A)** and phyla **(B)** in the Benign and CA groups. **(C)** Covariate bubble charts comparing the relative abundance of key differential taxa between groups, with circle size representing relative abundance and color denoting taxonomic clades. **(D)** Spearman correlation network displaying relationships between the abundance of core bacterial genera and clinical covariates, including age, tumor stage, smoking history, and molecular markers (p16 and Ki67).

At the phylum level, both the CA and Benign groups were dominated by Firmicutes and Bacteroidetes, followed by Proteobacteria and Actinobacteria (**Figure 3B**). However, significant differences in relative abundance were observed between the two groups: Firmicutes and Proteobacteria exhibited elevated relative abundance in the CA group, while Bacteroidetes and Actinobacteria were more prominent in the Benign group. At the genus level, the distribution of the top 10 dominant genera revealed compositional differences between groups (**Figure 3A**). *Streptococcus*, *Escherichia/Shigella*, and *Megamonas* showed increased relative abundance in the CA group, whereas *Bacteroides*, *Prevotella*, *Bifidobacterium*, and Ruminococcus were more abundant in the Benign group. Bubble plots further illustrated the abundance distribution of major differential genera, revealing significant enrichment of certain genera in the CA group (e.g., *Streptococcus* and Escherichia/Shigella), while *Prevotella* and *Bacteroides* were more prevalent in the Benign group (**Figure 3C**). In the correlation analysis between genus-level abundance and clinical characteristics, heatmap results revealed that multiple genera were significantly associated with patient age, gender, tumor stage, smoking history, and immunohistochemical markers (p16, Ki67) (**Figure 3D**). For example, *Streptococcus* showed a positive correlation with tumor stage, while *Bacteroides* exhibited a negative correlation with smoking history. Stage-stratified community profiles at the phylum and genus levels, and genus levels differential bubble charts, are provided in **Supplementary Figures 2A–C** and support the above patterns across Benign, EarlyLSCC, and LateLSCC. These observations highlight the distinct taxonomic distribution patterns and microbial shifts associated with laryngeal squamous cell carcinoma and its clinical progression.

### Differential bacterial genus analysis

**Figure 4:**
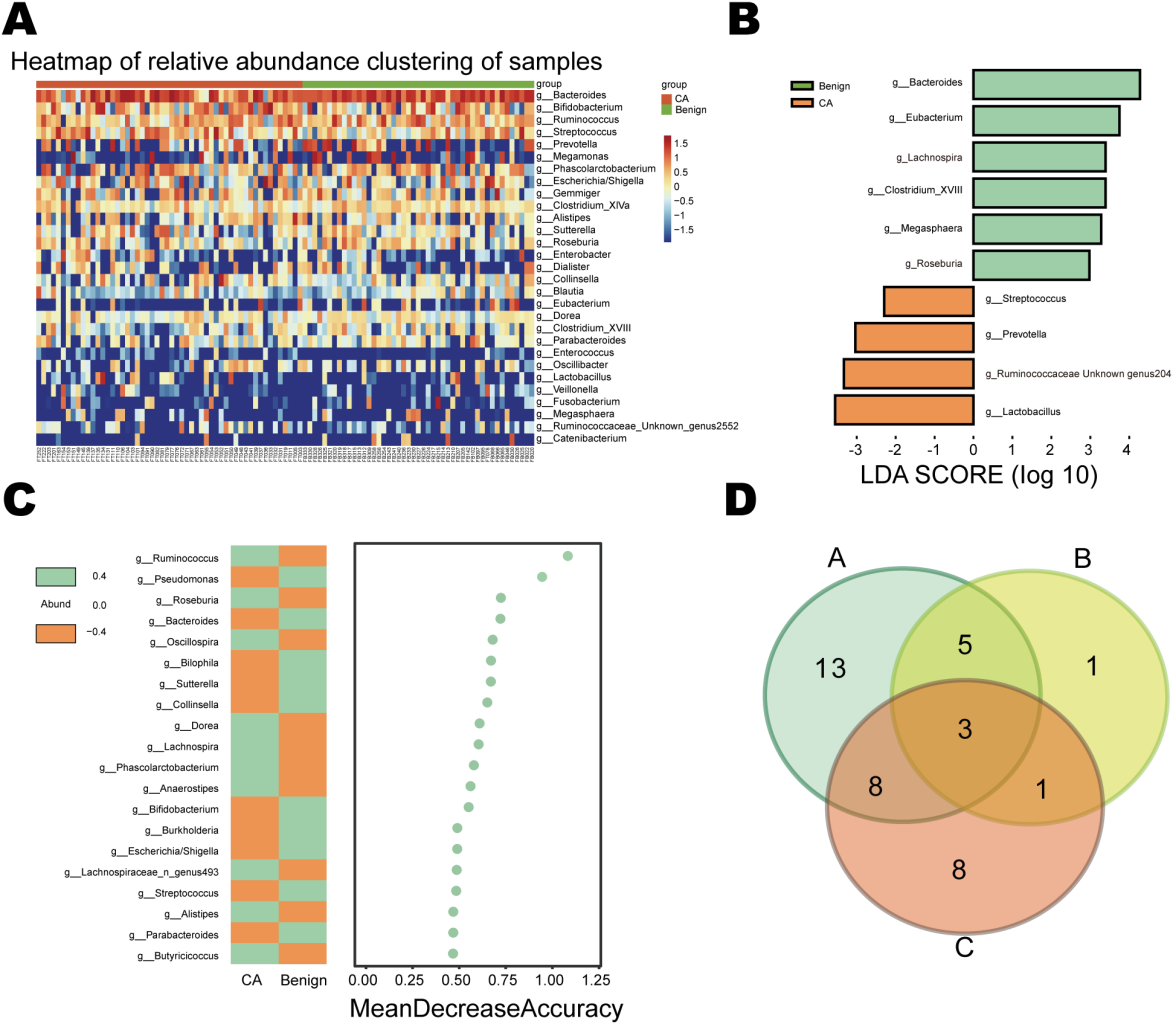
Identification of differentially abundant bacterial genera between Benign and LSCC groups. **(A)** Clustering heatmap of relative abundance for the most contributory bacterial genera across samples. **(B)** Linear discriminant analysis (LDA) effect size (LEfSe) identifying significantly enriched taxa in Benign (green) and CA (orange) groups (LDA score > 2.5). **(C)** Random Forest importance plot (Mean Decrease Accuracy) ranking the top 20 discriminating bacterial features between groups. **(D)** Venn diagram illustrating the intersection of core candidate genera identified by Wilcoxon rank-sum test **(A)**, LEfSe **(B)**, and Random Forest **(C)**.

To clarify compositional differences between Benign and LSCC, we employed multiple methods for cross-validation at the genus level. First, the clustering heatmap revealed distinct separation between Benign and CA samples, indicating systematic differences in community structure (**Figure 4A**). The Wilcoxon rank-sum test revealed enrichment of *Bacteroides*, *Eubacterium*, *Lachnospira*, Clostridium XVIII, *Megasphaera*, and *Roseburia* in Benign, while *Streptococcus*, *Prevotella*, Ruminococcaceae[unclassified], and *Lactobacillus* were elevated in CA (**Figure 4B**). LEfSe analysis further validated these differences, with multiple genera showing significant enrichment in either CA or Benign groups (LDA > 2.5, p < 0.05). Random forest identified Ruminococcus, *Roseburia*, *Bacteroides*, *Oscillospira*, and *Sutterella* as highly contributory genera among the top 20 discriminating features (**Figure 4C**). Intersecting the results from the three methods yielded 17 consistent candidate genera, including *Streptococcus*, *Bacteroides*, and *Roseburia*, which exhibited highly consistent distribution patterns across Benign and LSCC (**Figure 4D**). Stratified analysis revealed that these differences intensified or shifted with disease progression: clustering, LEfSe, and random forest results at the genus level were consistent across the three groups (Benign, EarlyLSCC, LateLSCC) (**Supplementary Figures 2D–F**). These findings suggest that these bacterial genera may serve as key microbial biomarkers for laryngeal carcinoma development.

### Stable genus screening and diagnostic modeling

**Figure 5:**
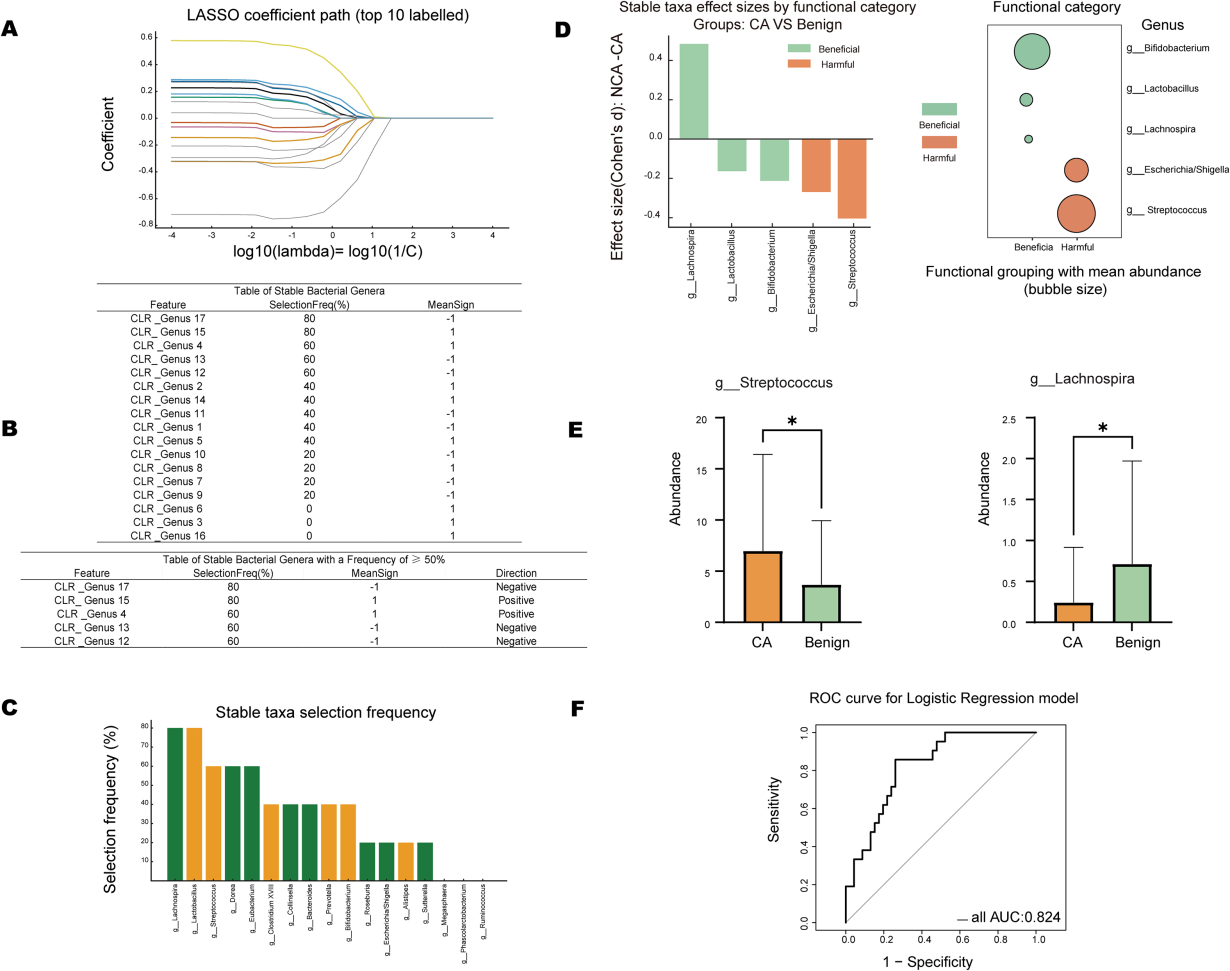
Construction and evaluation of the microbial-based diagnostic model. **(A)** LASSO coefficient path plot illustrating the selection of stable bacterial features via nested cross-validation. **(B)** Tables listing the stable bacterial genera with a selection frequency ≥ 50% and their coefficient directionality. **(C)** Bar chart showing the selection frequency of stable taxa during modeling. **(D)** Effect size (Cohen’s d) of stable genera categorized by their functional roles (Beneficial vs. Harmful). **(E)** Boxplots comparing the abundance of Streptococcus and Lachnospira between groups (*P < 0.05). **(F)** Receiver Operating Characteristic (ROC) curve showing the diagnostic performance of the logistic regression model built with stable genera (AUC = 0.824).

Using LASSO regression combined with repeated sampling validation, several bacterial genera with stable discriminative power between Benign and LSCC were identified (**Figures 5A–C**). Among these, *Lactobacillus*, *Streptococcus*, and *Escherichia/Shigella* showed positive correlations in the LSCC groups, while *Lachnospira* and *Bifidobacterium* were relatively enriched in the Benign group. Genera with screening frequencies ≥50% were defined as “stable genera,” yielding five core candidate genera. Further analysis revealed that these stable genera could be functionally categorized based on their reported roles in prior literature as potential probiotic-associated genera (e.g., *Lachnospira*, *Bifidobacterium*, *Lactobacillus*) and potential pathogen-associated genera (e.g., *Streptococcus*, *Escherichia/Shigella*) (**Figure 5D**). The observed dysbiosis was characterized by a concerted shift: the expansion of opportunistic pathogens, specifically *Streptococcus* and *Escherichia/Shigella*, in the LSCC groups, alongside the marked depletion of beneficial, butyrate-producing taxa such as *Lachnospira* and *Bifidobacterium* in the Benign group. Although their specific biological impact in the context of LSCC requires further experimental validation, differential analysis confirmed that *Streptococcus* was significantly enriched in the LSCC groups, whereas *Lachnospira* was more abundant in the Benign group (**Figure 5E**). A logistic regression model constructed based on these stable genera demonstrated high diagnostic efficacy in distinguishing Benign from malignant lesions, achieving an area under the ROC curve (AUC) of 0.824 (**Figure 5F**). Stratified analysis revealed that this model demonstrated superior discriminatory power in distinguishing Benign lesions from LateLSCC patients, suggesting its preliminary potential for clinical stratification, pending validation in broader, independent cohorts.

### Functional prediction

**Figure 6:**
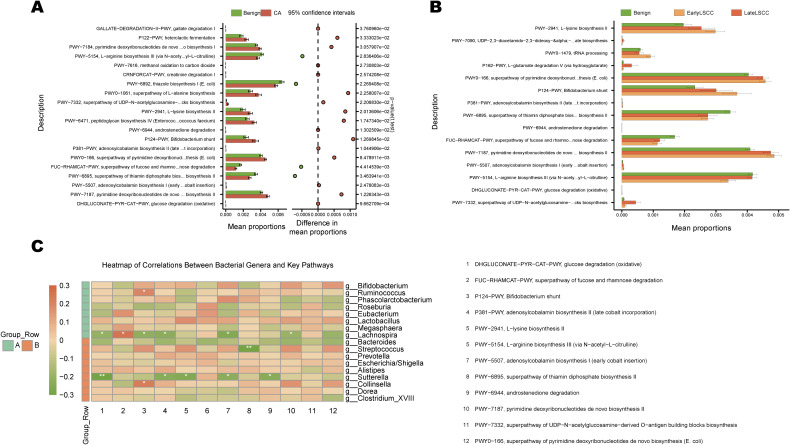
Functional pathway imbalances inferred by PICRUSt2. **(A)** Bar chart showing mean proportions and significant differences in metabolic pathways between Benign (green) and CA (red) groups. **(B)** Stage-specific comparison of pathway proportions across Benign, EarlyLSCC, and LateLSCC groups, highlighting metabolic shifts during disease progression. **(C)** Heatmap of Spearman correlations between the abundance of stable bacterial genera and key metabolic pathways, identifying specific microbes associated with metabolic remodeling.

Functional prediction based on PICRUSt2 indicates that the cancerous group (CA) exhibits enrichment in multiple pathways associated with cell wall and amino acid synthesis, including L-lysine biosynthesis (PWY-2941), peptidoglycan biosynthesis (e.g., PWY-6471), and the UDP-N-acetylglucosamine neosynthesis superpathway (PWY-7332). This was accompanied by upregulation of pathways related to nucleotide synthesis (e.g., the pyrimidine/purine pathway superpathway, PWY-0166, and PWY-7187/7184) (**Figure 6A**). In contrast, the Benign group showed greater enrichment in pathways related to vitamin/coenzyme synthesis and steroid metabolism, including adenosylcobalamin (vitamin B12) synthesis (PWY-5507/-P381), thiamine diphosphate biosynthesis (PWY-6895), and androstenedione degradation (PWY-6944), with significant upregulation of the *Bifidobacterium* shunt (P124-PWY) (**Figure 6A**). Stage-specific analysis revealed that the vast majority of differentially regulated pathways were already significantly altered in EarlyLSCC, with overall plateauing or decline as disease progressed; However, glutamate degradation via the hydroxyglutarate pathway (P162-PWY) further increased in late stages, suggesting deepening metabolic reprogramming during progression (**Figure 6B**). Microbiota-function association analysis revealed positive correlations between Escherichia/Shigella and *Streptococcus* with nucleotide synthesis and LPS/O-antigen pathways; *Bifidobacterium* and its characteristic *Bifidobacterium* shunt showed positive correlations; multiple butyrate/propionate-producing genera (e.g., *Roseburia*, *Lachnospira*) exhibited inverse associations with vitamin B and carbohydrate metabolism pathways (**Figure 6C**). In summary, a functional imbalance characterized by “increased cell wall/amino acid synthesis and decreased vitamin/steroid metabolism” exists between laryngeal cancer and Benign lesions. Most differences emerge in the early stages, with certain amino acid degradation pathways further intensifying in the late stages. This supports the hypothesis that “gut microbiota-mediated metabolic remodeling” contributes to the initiation and progression of laryngeal cancer.

## Discussion

### Independent association between gut microbiota dysbiosis and LSCC

This study systematically reveals for the first time characteristic alterations in the gut microbiota of patients with laryngeal squamous cell carcinoma (LSCC) at the fecal level. Although no significant differences in α diversity were observed, β diversity indicated distinct separation of community structures, with remodeling evident at both taxonomic and functional levels. The LSCC groups exhibited enrichment of *Streptococcus* and *Lactobacillus*, whereas the Benign group showed enrichment of butyrate-producing bacteria such as *Bacteroides* and *Lachnospira*. Functional prediction further suggested potential metabolic remodeling, wherein metabolic activities in the LSCC groups appeared to favor cell wall and amino acid synthesis, while the Benign group favored vitamin and steroid metabolism (19). These findings indicate that gut microbiota dysbiosis is significantly associated with the presence of LSCC. Regarding the potential confounding effect of age, it is well-documented that the gut microbiome undergoes significant shifts during aging, typically characterized by decreased diversity and a relative increase in pro-inflammatory commensals. However, several lines of evidence support the LSCC-specificity of our identified microbial signatures. First, while a statistically significant age gap existed between the Benign and LateLSCC groups (P = 0.019), we employed multivariate linear models (e.g., MaAsLin2) and PERMANOVA that included age as a covariate. This statistical adjustment allows for the isolation of the disease effect from age-related variance. Second, the specific enrichment of Streptococcus and the depletion of butyrate-producing Lachnospira observed in our LSCC cohort are distinct from generalized aging patterns reported in healthy elderly populations, which often center on different taxa such as Akkermansia or specific Bacteroides shifts. Thus, the microbial remodeling identified here likely reflects the unique systemic metabolic and immune pressures exerted by laryngeal malignancy rather than mere chronological aging. Given the cross-sectional nature of this study, these results represent a significant association rather than a causal relationship, and we cannot clarify the temporal sequence between microbiome remodeling and laryngeal tumorigenesis. Furthermore, while the identified gut signatures appear distinct, future comparative studies involving simultaneous sampling of the oral and respiratory microbiota are necessary to confirm the relative independence of these microbial niches.

### Evidence extending to the “gut-organ axis”

Previous evidence primarily focused on gastrointestinal tumors (24). However, recent studies indicate that gut microbiota can influence distant organs—including brain, liver, breast, and lung tumors—through immune, metabolic, and inflammatory regulation (10, 11, 25–27). These findings support the role of the “gut-organ axis” in the development of systemic malignancies. Our findings reveal that malignant tumors in the larynx—a site distant from the gut—exhibit characteristic gut microbiota dysbiosis. This not only broadens the scope of the microbiome-cancer relationship but also suggests that gut microbiota may potentially function as an important systemic factor associated with the landscape of carcinogenesis.

### Potential mechanisms

Our findings suggest that the gut microbiome restructuring in LSCC is driven by a dual-action dynamic of pathogenic expansion and probiotic depletion. The significant enrichment of *Streptococcus* and *Escherichia/Shigella* in LSCC patients aligns with the “expansion of opportunistic pathogens” model. These taxa are often associated with increased lipopolysaccharide (LPS) production and pro-inflammatory signaling, which our functional prediction also highlighted as potential metabolic remodeling through the inferred upregulation of cell wall and nucleotide synthesis pathways. Conversely, the “depletion of beneficial taxa” is evidenced by the loss of *Lachnospira* and *Bacteroides* in the LSCC. As primary producers of short-chain fatty acids (SCFAs), their reduction may result in a compromised intestinal barrier and diminished systemic anti-inflammatory capacity, potentially favoring a systemic environment conducive to tumor progression.

Functional dysregulation provides clues to the underlying mechanisms. On one hand, enriched bacterial genera such as *Streptococcus* may create a pro-cancerous environment through lactic acid metabolism, mucosal inflammation, and barrier disruption (28, 29). On the other hand, reduced butyrate-producing bacteria and downregulated vitamin metabolism may compromise immune homeostasis, facilitating immune evasion (29). Concurrently, the predicted upregulation of cell wall and amino acid synthesis suggests potential metabolic remodeling, accelerating tumor progression. These mechanisms align strongly with prior observations in colorectal and liver cancers, supporting the microbiota–metabolism–immune interaction as a critical pathway in LSCC development.

### Preliminary prospects for clinical application

Our multi-method screening and LASSO regression-derived stable differentially abundant genera enabled the establishment of a diagnostic model demonstrating excellent performance in distinguishing benign from malignant lesions, particularly in LateLSCC (30). This suggests that fecal microbiota signatures hold potential as candidates for non-invasive biomarkers for diagnosis and staging; however, their diagnostic robustness must be rigorously tested across geographically and demographically diverse populations before any clinical implementation can be considered (31, 32). Concurrently, alterations in functional pathways provide a theoretical basis for intervention: through dietary modifications, probiotic supplementation, or targeted metabolite therapy, microbiome regulation may be achievable, potentially playing a role in prevention and adjuvant treatment (33).

### Limitations and outlook

A primary limitation of this study is the relatively small sample size, particularly within the LateLSCC subgroup (N = 21). While the overall cohort (N = 101) provided sufficient power for the primary comparison between Benign and Malignant groups, the reduced size of the late-stage sub-cohort significantly constrains the statistical power to detect more subtle, stage-specific microbial shifts. Consequently, it is possible that certain biologically relevant but less pronounced microbial differences between early and late stages remained undetected. Therefore, our findings regarding stage-specific markers must be interpreted as exploratory and preliminary. These results should be viewed as a foundation for future, better-powered longitudinal studies rather than definitive evidence of stage-specific dysbiosis patterns. Additionally, while efforts were made to minimize variance by sampling during hospitalization with standardized meals, several critical confounding factors were not fully addressed. Factors such as Body Mass Index (BMI), pre-existing comorbidities (e.g., metabolic syndrome), and long-term use of non-antibiotic medications (e.g., proton pump inhibitors or metformin), which are known to shape the gut microbiome, were not systematically analyzed in this retrospective cohort. The lack of detailed long-term dietary logs further limits our ability to exclude the impact of chronic dietary patterns. Future studies should incorporate these variables into multivariate or matching models to refine the independent association between the gut microbiome and LSCC. Furthermore, due to the relatively limited follow-up period, long-term prognostic and survival data were not available, which precluded the performance of Cox multivariate regression analysis to evaluate the impact of the gut microbiota on patient outcomes. Future longitudinal studies with extended follow-up durations are essential to determine whether these microbial signatures can serve as reliable predictors of clinical prognosis in LSCC. Regarding the clinical utility of our diagnostic model, caution is warranted. Given that the model was built on a single-center cohort, the current performance metrics might be overly optimistic despite the 100-repeated nested cross-validation (Nested CV). Substantial independent, multi-center validation in diverse populations is an absolute requirement before these microbial signatures and the diagnostic model can be considered for practical clinical application. Finally, 16S rRNA sequencing analysis did not cover fungi, the virome, or the metabolome (34). Additionally, the functional pathways reported here were inferred via PICRUSt2 rather than being directly measured through direct metabolomics. It is crucial to clarify that these results represent potential metabolic remodeling rather than confirmed enzymatic activity. While these predictions provide valuable hypothetical insights, they lack direct mechanistic validation and should be interpreted with caution.

## Conclusion

This study confirms that both the overall structure and functional pathways of the gut microbiota undergo remodeling in laryngeal cancer compared to Benign lesions. Pathogenic-associated genera such as *Streptococcus* and *Lactobacillus* are enriched in malignant samples, while *Bacteroides*, *Lachnospira*, and *Roseburia* dominate in Benign samples. Core genera identified through multi-method cross-validation and LASSO stable screening (including g_*Streptococcus*, g_*Lachnospira*, g_*Lactobacillus*, g_*Bifidobacterium*, g_*Escherichia/Shigella*) robustly distinguish between groups. From the perspective of potential metabolic remodeling (rather than confirmed enzymatic activity), functional predictions suggested that malignancy favored cell wall and amino acid synthesis pathways, while Benign cases leaned toward vitamin coenzyme and steroid metabolism; most differences emerged early. Models built with stable differential bacteria performed well in distinguishing “Benign vs. LateLSCC,” suggesting their exploratory potential as candidates for non-invasive biomarkers and early risk stratification tools. These findings serve as a preliminary proof-of-concept that requires confirmation through robust, multi-center prospective trials. While the present findings suggest a potential role for the gut microbiome in LSCC, their clinical application for intervention remains hypothetical. These results provide a foundation for future research into microbial modulation and non-invasive diagnostics, though rigorous mechanistic validation and longitudinal trials are required before any clinical translation can be considered.

## Data Availability

The datasets presented in this study can be found in online repositories. The names of the repository/repositories and accession number(s) can be found in the article/**Supplementary Material**.
