# Supplementary figures and images for: Gut microbiome restructuring in laryngeal squamous cell carcinoma identifies stable microbial biomarkers with diagnostic potential
Source: Front Oncol. 2026 Apr 22;16:1788705. doi: 10.3389/fonc.2026.1788705 (PMC13143588; doi:10.3389/fonc.2026.1788705)

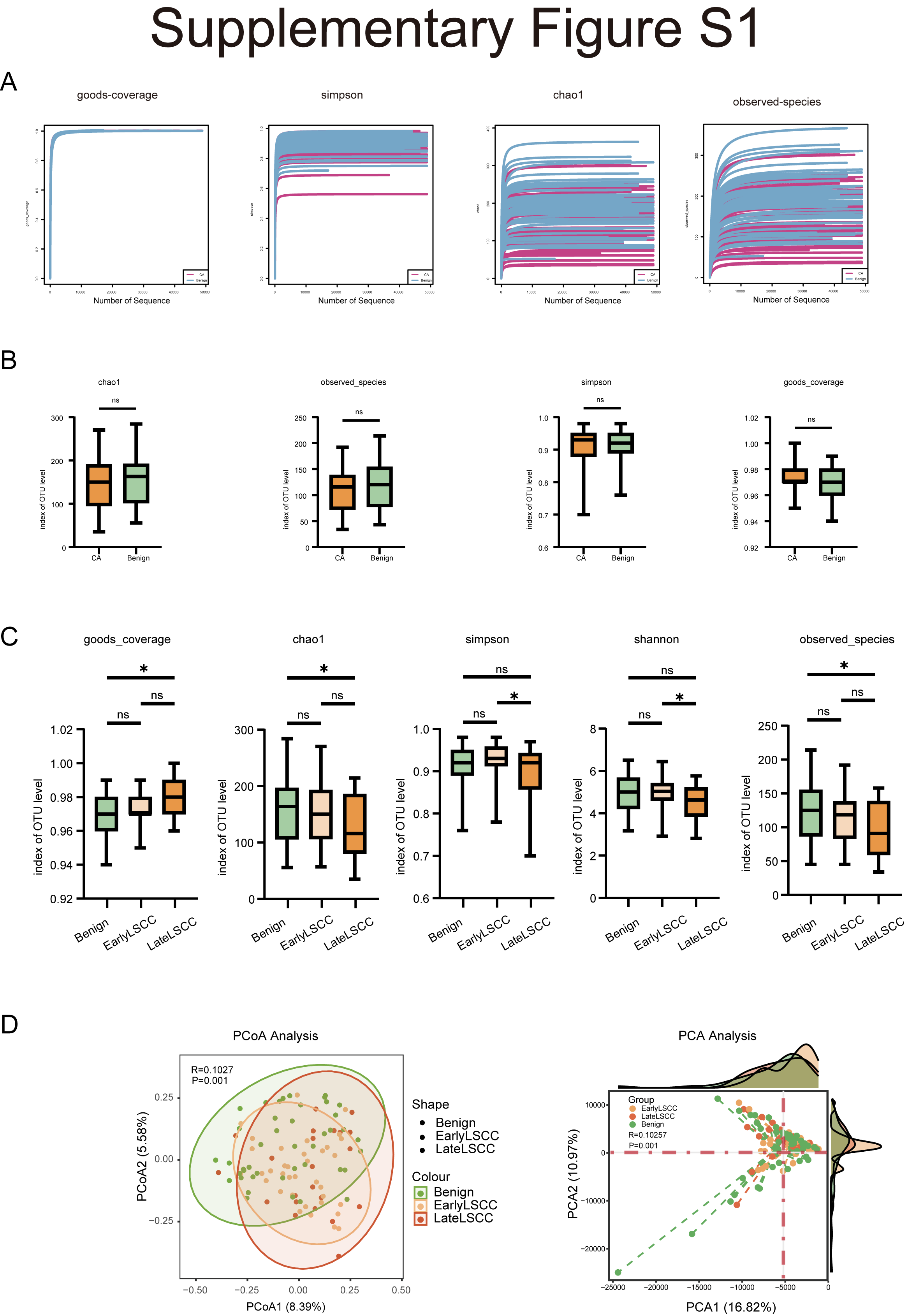

Supplement: Supplementary file 1 [file Image1.tif]

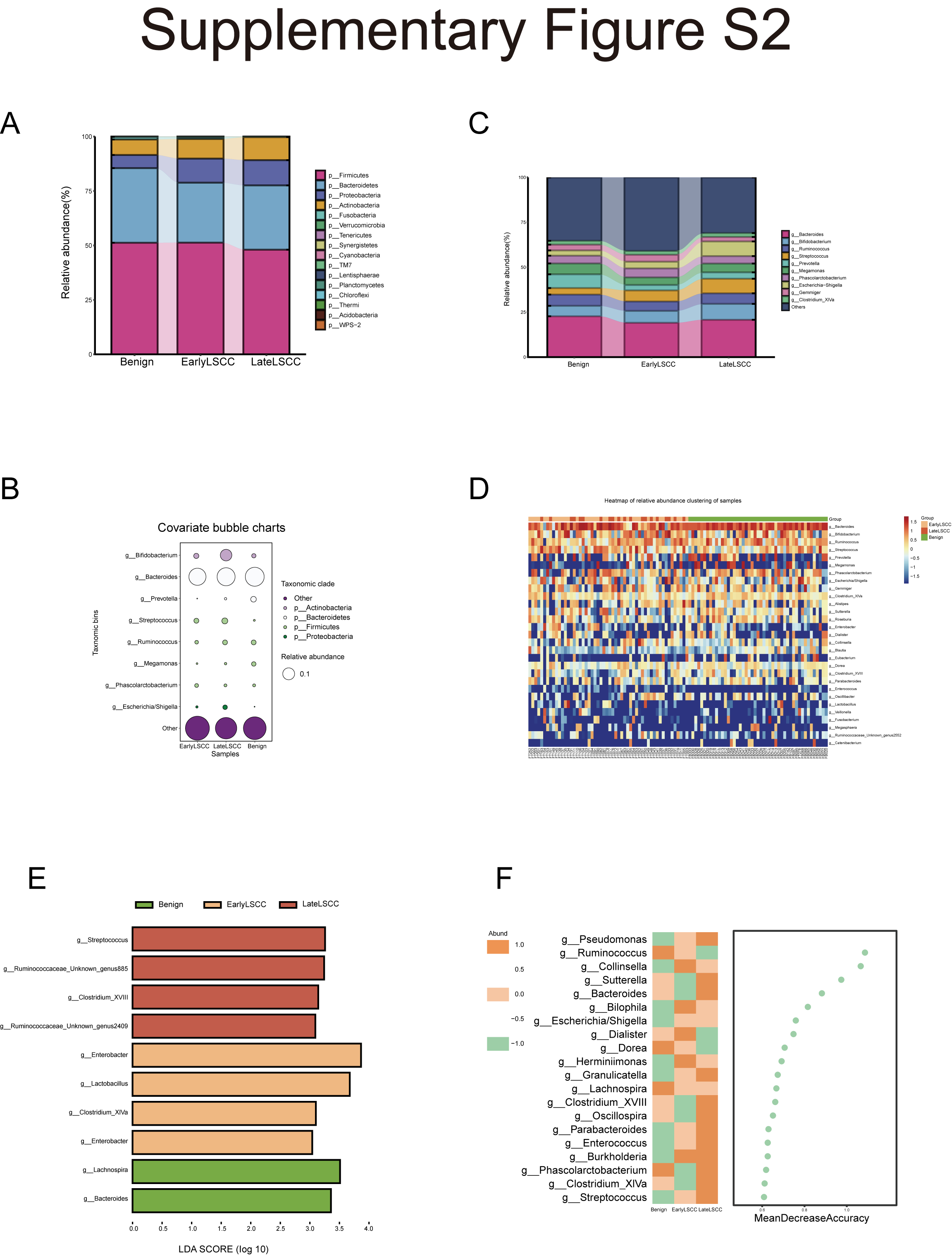

Supplement: Supplementary file 2 [file Image2.tif]

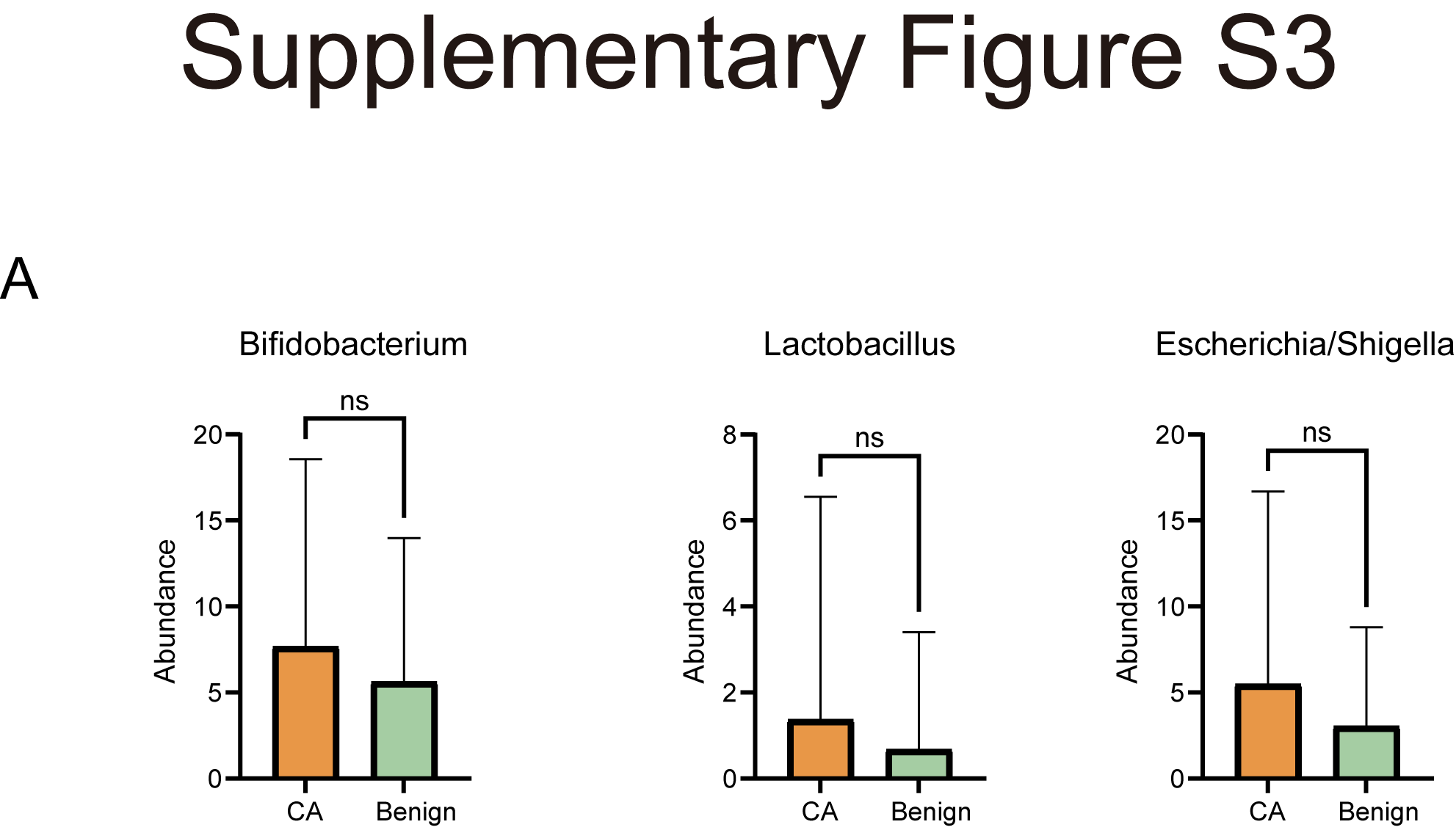

Supplement: Supplementary file 3 [file Image3.tif]
